# ETHNORACIAL DIFFERENCES IN HOME HEALTHCARE USE: FINDINGS FROM THE NATIONAL HEALTH AND RETIREMENT STUDY

**DOI:** 10.1093/geroni/igac059.1860

**Published:** 2022-12-20

**Authors:** Adam Simning, Jinjiao Wang, Thomas Caprio, Brian McGarry, Yue Li

**Affiliations:** University of Rochester, Rochester, New York, United States; University of Rochester School of Nursing, Rochester, New York, United States; University of Rochester, Rochester, New York, United States; University of Rochester, Rochester, New York, United States; University of Rochester, Rochester, New York, United States

## Abstract

This study seeks to identify ethnoracial differences in self-reported home health care (HHC) use among older adults. To do so, we examined 8,817 people aged 65 and older from the 2016 wave of the Health and Retirement Study (HRS), a nationally representative survey of older adults in the U.S. The dependent variable was whether HRS participants reported any HHC service use in the past two years. The primary independent variable was ethnoracial grouping, which included non-Hispanic White, non-Hispanic Black, and Hispanic groups. Multivariable logistic regressions stratified by ethnoracial grouping identified correlates of HHC use. We found that HHC use was more prevalent among non-Hispanic Blacks (14.9%) than in non-Hispanic Whites (10.1%) or Hispanics (10.7%). For all ethnoracial groups, increasing age, dementia, activities of daily living impairment, medical comorbidity (except for Hispanics), and hospitalization (in the past two years) were associated with an increased likelihood of HHC use. In addition, we identified ethnoracial differences in the correlates of HHC use. Among non-Hispanic Whites, more formal education and Medicaid insurance were associated with a higher likelihood of using HHC. For non-Hispanic Blacks, residing in rural areas was associated with a decreased likelihood of HHC use, whereas being single and living alone were associated with an increased likelihood of HHC use. This study thereby identified notable ethnoracial differences in the correlates of HHC use among older adults.

